# BMC *Musculoskeletal Disorders* reviewer acknowledgement 2014

**DOI:** 10.1186/s12891-015-0463-z

**Published:** 2015-02-07

**Authors:** Catia Cornacchia

**Affiliations:** BioMed Central, Floor 6, 236 Gray’s Inn Road, London, WC1X 8HB United Kingdom

**Ellen Aartun**

Denmark

**Joseph Abboud**

USA

**Christina Abdel Shaheed**

Australia

**Adham Abdelrahim**

USA

**Paul Ackermann**

Sweden

**Raphael Adobor**

Norway

**Abbas Agaimy**

Germany

**Yong Ahn**

Korea, South

**Philipp Ahrens**

Germany

**Dawn Aitken**

Australia

**Maassoumeh Akhlaghi**

Iran

**Esin Aktas Cetin**

Turkey

**Nuray Aktay Ayaz**

Turkey

**Marta Alarcón-Riquelme**

Sweden

**Hassan Albadawi**

USA

**Francisco Alburquerque-Sendín**

Spain

**Tiago Alexandre**

Brazil

**Omid Aliadehkhaiyat**

UK

**Kelli Allen**

USA

**Kyle Allen**

USA

**Amer Almohallami**

Germany

**Tjarco D.W. Alta**

Netherlands

**Saad Altaher**

Saudi Arabia

**Stephen Alway**

USA

**Paola Amable**

Brazil

**Koichi Amano**

Japan

**Michael Amling**

Germany

**Corinne Ammann-Reiffer**

Switzerland

**Kenneth Jay Andersen**

Denmark

**Lars L. Andersen**

Denmark

**Tonny Andersen**

Denmark

**Sanjeev Aneja**

India

**Naffis Anjarwalla**

UK

**La Torre Antonio**

Italy

**Samuel Antwi-Baffour**

Ghana

**A. Vania Apkarian**

USA

**Adamantios Arampatzis**

Germany

**Nigel Arden**

UK

**Albert Arman Riera**

Spain

**Toni Arndt**

Sweden

**Asbjørn Årøen**

Norway

**Hiroshi Asahara**

Japan

**Ali Asghari**

Iran

**Robert Ashford**

UK

**Henry Dushan Edward Atkinson**

UK

**Isam Atroshi**

Sweden

**Cathrine Austad**

Norway

**Luke Austin**

USA

**Olufemi Ayeni**

Canada

**Keith Baar**

USA

**Ji-Hoon Bae**

Korea, South

**Saleh Baeesa**

Saudi Arabia

**Isabel Baert**

Belgium

**Carlos Bagley**

USA

**Maurice Balke**

Germany

**Elisabeth Ball**

UK

**Alejandro Balsa**

Spain

**Ingo Banke**

Germany

**Igor Baptista**

Brazil

**Mary Barbe**

USA

**Alexandre Barbosa**

Brazil

**Giancarlo Barolat**

USA

**Richard Barrett-Jolley**

UK

**Bernadette Bartlam**

UK

**Genevieve Baujat**

France

**Petra Bäumler**

Germany

**David Bazett-Jones**

USA

**Lindsay Bearne**

UK

**Jeferson Becker**

Brazil

**Michael Becker**

USA

**Yiftah Beer**

Israel

**Iain Beith**

UK

**Knut Beitzel**

Germany

**Francesco Benazzo**

Italy

**Kathleen Bennett**

Ireland

**Stefan Bergman**

Sweden

**Julien Berhouet**

France

**Jessica Bertrand**

Germany

**Edmundo Berumen-Nafarrate**

Mexico

**Elyssa Besen**

USA

**Josette Bettany-Saltikov**

UK

**Bryan Beutel**

USA

**Deepu Bhaskar**

UK

**Gerolamo Bianch**

Italy

**Roland Biber**

Germany

**Rudolf Billeter**

UK

**Trevor Birmingham**

Canada

**Annette Bishop**

UK

**Leanne Bisset**

Australia

**Tony Bjourson**

UK

**Jason Bleedorn**

USA

**Dana Bliuc**

Australia

**Benjamin Blondel**

France

**Gerrit Bode**

Germany

**Richard Bohannon**

USA

**Megan Bohensky**

Australia

**Harald Böhm**

Germany

**Matthieu Boisgontier**

Belgium

**Nils Bömer**

Netherlands

**Jorge Boretto**

Argentina

**Cory Borkhoff**

Canada

**Judith Bosmans**

Netherlands

**Daniel Bossen**

Netherlands

**Samy Bouaicha**

Switzerland

**Karim Bougatef**

Tunisia

**Judith V.M.G. Bovée**

Netherlands

**Catherine Bowen**

UK

**Frank Braatz**

Germany

**Julii Brainard**

UK

**Thomas Viskum Gjelstrup Bredahl**

Denmark

**Roelf Breederveld**

Netherlands

**Alan Breen**

UK

**Leigh Breen**

UK

**Sharon Brennan**

Australia

**Jane Brice**

USA

**Andrew Briggs**

Australia

**James Browne**

USA

**Julie Bruce**

UK

**Adam Bryant**

Australia

**Stefan Buchmann**

Germany

**Maciej Buchowski**

USA

**Richard Buckley**

Canada

**Conor Buckley**

Ireland

**Jenni Buckley**

USA

**Cody Bünger**

Denmark

**Alex Burdorf**

Netherlands

**Paul Burgers**

Netherlands

**Gerard Bury**

Ireland

**Johannes Bussmann**

Netherlands

**Martin Buttaro**

Argentina

**Mario Cabraja**

Germany

**Leonard Calabrese**

USA

**Joaquin Calatayud**

Spain

**Giorgio Maria Calori**

Italy

**Anna Campanati**

Italy

**Lorna Campbell**

UK

**Paul Campbell**

UK

**Stefano Campi**

Italy

**Helena Canhao**

Portugal

**Mark A. Carlson**

USA

**F. David Carmona**

Spain

**Antonio Caronni**

Italy

**Christopher Carty**

Australia

**Christian Carulli**

Italy

**Matteo Castaldo**

Italy

**Antonino Catalano**

Italy

**Giovanna Cenacchi**

Italy

**Jacqueline Center**

Australia

**Bp Chan**

China

**Danny Chan**

China

**Deva Chan**

USA

**Vinod Chandran**

Canada

**Shun-Jen Chang**

Taiwan

**Moon Jong Chang**

Korea, South

**Boqing Chen**

USA

**Dafu Chen**

China

**Yi-Xin Chen**

China

**Neal Chen**

USA

**Huajiang Chen**

China

**Suzan Chen**

Canada

**Wen-Shiang Chen**

Taiwan

**Wei Ming Chen**

Taiwan

**Weng-Pin Chen**

Taiwan

**Chih-Hsiu Cheng**

Taiwan

**Chia-Ho Cheng**

USA

**Xiao-Guang Cheng**

China

**Yung-Hsiao Chiang**

Taiwan

**Eugenio Chiarello**

Italy

**Alessandro Chiarotto**

Netherlands

**Ko Chiba**

Japan

**Nachiappan Chockalingam**

UK

**Hyuk Choi**

Korea, South

**Jacek Cholewicki**

USA

**William Chong**

USA

**Dean Chou**

USA

**Wen-Chi Chou**

USA

**Nikolaos Christidis**

Sweden

**Panayiotis Christofilopoulos**

Switzerland

**Elisabeth Chroni**

Greece

**Flavia Cicuttini**

Australia

**Cara Cipriano**

USA

**Nicholas Clarke**

UK

**Alberto Cliquet Jr**

Brazil

**Laura Coates**

UK

**David Coggon**

UK

**Juan C Colado**

Spain

**Sascha Colen**

Belgium

**Richard Conway**

Ireland

**Brooke Coombes**

Australia

**Michel Coppieters**

Netherlands

**Mark Corbett**

UK

**Divi Cornec**

France

**Mark Cote**

USA

**Christian Couppé**

Denmark

**Charles Court-Brown**

UK

**Laura Creemers**

Netherlands

**Richard Crist**

USA

**Peter Croft**

UK

**Antonio Ignacio Cuesta-Vargas**

Australia

**David Culliford**

UK

**Randall Culp**

USA

**Tara Cusack**

Ireland

**Marcus Czabanka**

Germany

**Danè Dabirrahmani**

Australia

**Jin Dai**

China

**Raffaele D'Amelio**

Italy

**Gali Dar**

USA

**Nancy Daraiseh**

USA

**Amitabh Dashottar**

USA

**John Davis**

USA

**Jennifer Davis**

Canada

**Eling D. De Bruin**

Switzerland

**Massimo De Filippo**

Italy

**Silvana De Giorgi**

Italy

**Fernanda De Lima E Sa Resende**

Italy

**Katie De Luca**

Australia

**Maarten De Wit**

Netherlands

**Masataka Deie**

Japan

**Eamonn Delahunt**

Ireland

**Edgard Delvin**

Canada

**Andreas K Demetriades**

UK

**Christophe Demoulin**

Belgium

**Alfons Den Broeder**

Netherlands

**Dennis Den Hartog**

Netherlands

**Jessica Deneweth**

USA

**Sheena Derry**

UK

**Patrick Dessein**

South Africa

**Ryan Dewall**

USA

**Luca Di Geso**

Italy

**Michelino Di Rosa**

Italy

**Luis Diambra**

Argentina

**Guoqing Diao**

USA

**Jose Diaz-Coto**

Costa Rica

**Thomas Dienstknecht**

Germany

**Paul Dieppe**

UK

**Umit Dincer**

Turkey

**Guanxiong Ding**

China

**Qiang Ding**

China

**Amelia Dobbins**

Australia

**Rachael Docking**

UK

**Leslie Dodd**

USA

**Mieke Dolphens**

Belgium

**Davide Maria Donati**

Italy

**Simon Donell**

UK

**Qi Rong Dong**

China

**James Dowling**

Canada

**Thomas Dreher**

Germany

**Karsten Dreinhoefer**

Germany

**Jean-Daniel Dubois**

Canada

**Neil Duncan**

Canada

**Kate Dunn**

UK

**Mona Dvir-Ginzberg**

Israel

**Krysia Dziedzic**

UK

**Perajit Eamsobhana**

Thailand

**Arul Earnest**

Australia

**Ingrid Eitzen**

USA

**Yakup Ekinci**

Turkey

**Richard Ekstrom**

USA

**Maha El Gaafary**

Egypt

**Omar El-Daly**

Egypt

**Nurzat Elmali**

Turkey

**David Elson**

UK

**Bilal Farouk El-Zayat**

Germany

**Martin Englund**

Sweden

**Anders Enocson**

Sweden

**Paul Enthoven**

Sweden

**Markus Ernst**

Switzerland

**Ali Ersen**

Turkey

**Antonio Escobar**

Spain

**Ansgar Espeland**

Norway

**Daniel Espino**

UK

**Jean-Pierre; Christian Estebe**

France

**Evangelos Evangelou**

Greece

**Angela Evans**

Australia

**Andrea Evers**

Netherlands

**Ayman Fahim**

United Arab Emirates

**Deborah Falla**

Germany

**Omar Faour Martin**

Spain

**Farzam Farahmand**

Iran

**Andrea Farioli**

Italy

**Nicholas Farmer**

UK

**Francis Fatoye**

UK

**Eugen Feist**

Germany

**Shiqing Feng**

China

**Cesar Fernandez-De-Las**

Spain

**Mariano Fernandez-Fairen**

Spain

**Manuela Ferreira**

Australia

**Emmanuelle Ferrero**

France

**Giorgio Ferriero**

Italy

**Gianluca Ficca**

Italy

**Danilo Fintini**

Italy

**Antonella Fioravanti**

Italy

**Diarmaid Fitzgerald**

Ireland

**James Fitzsimmons**

USA

**Fernando Fonseca**

Portugal

**Sergio Fonseca**

Brazil

**Raimund Forst**

Germany

**Nicholas Robert Forsyth**

UK

**Lisa Fortier**

USA

**Nadine Foster**

UK

**Ulrich Fredberg**

Denmark

**Helen French**

Ireland

**Simon French**

Canada

**Antonio Frizziero**

Italy

**Takeshi Fuji**

Japan

**Naoto Fujita**

Japan

**Kenji Fujiwara**

Japan

**Naoshi Fukui**

Japan

**Philipp Funovics**

Austria

**Sebastian Fürderer**

Germany

**Victoria Furer**

USA

**Takayuki Furumatsu**

Japan

**Takefumi Furuya**

Japan

**C. Philip Gabel**

Australia

**Angelo Gaffo**

USA

**Axel Gamulin**

Switzerland

**Rajiv Gandhi**

Canada

**Zhichao Gao**

China

**Patric Garcia**

Germany

**Sara Garfield**

UK

**Nitin Garg**

India

**Adam Garrow**

UK

**Louie Gaston**

UK

**Angelica Gattamelata**

Italy

**Bart Geerts**

Netherlands

**Russell Gelfman**

USA

**Stephane Genevay**

Switzerland

**Paul Gerdhem**

Sweden

**Christian Gerhardt**

Germany

**Piet Geusens**

Netherlands

**Mabrouk Ghonaim**

Egypt

**Claudia Giacomozzi**

Italy

**Sandro Giannini**

Italy

**Peter Giannoudis**

UK

**Jorge Gil-Albarova**

Spain

**Karl Peter Gill**

UK

**Anne Gingery**

USA

**Karen Ginn**

Australia

**Dafna Gladman**

Canada

**Damjan Glavac**

Slovenia

**Ronald Glick**

USA

**Paritosh Gogna**

India

**Sara Gombatto**

USA

**Enrique Gomez-Barrena**

Spain

**Christoph Emanuel Gonser**

Germany

**Miguel A. Gonzalez-Gay**

Spain

**Rachael Gooberman-Hill**

UK

**Adam Goode**

USA

**Philip Goodney**

USA

**Jon Goosen**

Netherlands

**Markus Gosch**

Austria

**Christian Götze**

Germany

**Nikolaos Gougoulias**

UK

**Sibylle Grad**

Switzerland

**Susanne Grässel**

Germany

**Ed Greenfield**

USA

**Karen Greenwood**

USA

**Susan Grimston**

USA

**Kjersti Grønning**

Norway

**K. Gross**

USA

**Zarko Grozdanovic**

Germany

**Thorsten Guehring**

Germany

**Klaus-Peter Guenther**

Germany

**Boyko Gueorguiev**

Switzerland

**Enrique Guerado**

Spain

**Paulo Guerra**

Brazil

**Alberto Guevara-Alvarez**

Mexico

**Melek Guler-Yuksel**

Netherlands

**Ting Guo**

USA

**Zheng Guo**

China

**Shigong Guo**

UK

**Pratyush Gupta**

India

**Richa Gupta**

India

**Simona Gurzu**

Romania

**Gregory Gutierrez**

USA

**Gabriel Gutman**

Canada

**Diane Gyi**

UK

**Yong-Chan Ha**

Korea, South

**Tsjitske Haanstra**

Netherlands

**Mitchell Haas**

USA

**Florian Haasters**

Germany

**Yoshihiro Hagiwara**

Japan

**Yong Hai**

China

**Hildrun Haibel**

Germany

**Andrew Haig**

USA

**David Hak**

USA

**Matthew Halanski**

USA

**Amanda Hall**

UK

**Jill Halstead**

UK

**Harri Hamalaine**

Finland

**Peter Hamer**

Australia

**Talhatu Hammzat**

Nigeria

**Mark Hancock**

Australia

**Pascal Hannemann**

Netherlands

**Atsushi Harada**

Japan

**Helen Harcombe**

New Zealand

**Leila Harhaus**

Germany

**Kristina Harris**

UK

**Carisa Harris Adamson**

USA

**Blain Harrison**

USA

**Robert Hartman**

USA

**Bernd Hartmann**

Germany

**Jan Hartvigsen**

Denmark

**Masahiro Hasegawa**

Japan

**Daniël Haverkamp**

Netherlands

**Ziad Hawamdeh**

Jordan

**Gillian Hawker**

Canada

**Chengqi He**

China

**Xijing He**

China

**Emma Healey**

UK

**John Healey**

USA

**Yvonne Heerkens**

Netherlands

**Kurt Hegmann**

USA

**Behzad Heidari**

Iran

**Christian Heidemann**

Denmark

**Christian Heisel**

Germany

**Karl-Dieter Heller**

Germany

**Peter Helwig**

Germany

**Yves Henchoz**

Switzerland

**Paul Hendrick**

UK

**Marius Henriksen**

Denmark

**Marketta Henriksson**

Sweden

**Yves Henrotin**

Belgium

**Nicholas Henschke**

Germany

**Elizabeth Hensor**

UK

**Mirco Herbort**

Germany

**Georg Herget**

Germany

**Manfred Herold**

Austria

**Pablo Herrero**

Spain

**Matthew Herring**

USA

**Jay Hertel**

USA

**Lise Hestbaek**

Denmark

**Beat Hintermann**

Switzerland

**John Hipp**

USA

**Koji Hiraoka**

Japan

**Toshiaki Hirose**

Japan

**George Hirsch**

UK

**Michael Hirschmann**

Switzerland

**Anja Hirschmüller**

Germany

**Mei-Ling Ho**

Taiwan

**László Hodinka**

Hungary

**Alexander Hofmann**

Germany

**Kara Holloway**

Australia

**Joerg Holstein**

Germany

**Cathy Holt**

UK

**Boris Michael Holzapfel**

Australia

**Gerold Holzer**

Austria

**Maiko Hongo**

Japan

**Sittisak Honsawek**

Thailand

**Byron Hoogwerf**

USA

**Daniel Hoornenborg**

Netherlands

**Zdenek Horak Czech**

Republic

**Ru-Lan Hsieh**

Taiwan

**Shan-Hui Hsu**

Taiwan

**Jiang Hu**

USA

**Jianzhong Hu**

China

**Teng Huajian**

UK

**Chun-Yuh Huang**

USA

**Kui-Chou Huang**

Taiwan

**Kuo Feng Huang**

Taiwan

**Joerg Huber**

Switzerland

**Michaela K.I. Huber**

Germany

**Stefan Huber-Wagner**

Germany

**Cheryl Hubley-Kozey**

Canada

**Markus Huebscher**

Australia

**Sean Hughes**

UK

**Adwoa Hughes-Morley**

UK

**Sven Hungerer**

Germany

**Nguyen Huong**

Viet Nam

**Christof Hurschler**

Germany

**Vigdis Schnell Husby**

Norway

**Mozammil Hussain**

USA

**Charles Hutchinson**

UK

**Tiina Huusko**

Finland

**Anak Iamaroon**

Thailand

**Mazin Ibrahim**

UK

**Maria Inacio**

USA

**Ganesh Ingavle**

UK

**Adriana Ioachimescu**

USA

**Özgür Ismailoglu**

Turkey

**Hiromu Ito**

Japan

**Hiorshi Ito**

Japan

**Toshiki Iwase**

Japan

**Keisuke Izumi**

Japan

**Kenneth Izuora**

USA

**Fahimeh Jafarian**

Iran

**Holger Jahr**

Germany

**Nitin Jain**

USA

**Thomas Jakobsen**

Denmark

**Hendrik Jansen**

Germany

**Prawit Janwantanakul**

Thailand

**Jolanta Jaworek**

Poland

**Anis Jellad**

Tunisia

**Zsuzsa Jenei-Lanzl**

Germany

**Chris Jensen**

Denmark

**Tue Secher Jensen**

Denmark

**Xuemei Ji**

China

**Ching-Chuan Jiang**

Taiwan

**Qing Jiang**

China

**Xiaobing Jin**

USA

**Han Jinsong**

China

**Kitti Jirarattanaphochai**

Thailand

**Suenghwan Jo**

Korea, South

**Simon Jones**

UK

**Marjolein Jongbloed-Pereboom**

Netherlands

**Christoffer Jørgensen**

Denmark

**Andrew Judge**

UK

**Carsten Juhl**

Denmark

**Petro Julkunen**

Finland

**Zheng Junch**

China

**Kwang Am Jung**

Korea, South

**Noelle Junod Perron**

Switzerland

**Jesse Jupiter**

USA

**Anne Grethe Jurik**

Denmark

**Birgit Juul-Kristensen**

Denmark

**Yuho Kadono**

Japan

**Meldon Kahan**

Canada

**David Kahler**

USA

**Hiroshi Kaji**

Japan

**George Kalliolias**

USA

**Tunku Kamarul**

Malaysia

**Nobuhiro Kamiya**

USA

**Steven Kamper**

Australia

**Michael Kang**

USA

**Peter Kannu**

Canada

**Pekka Kannus**

Finland

**Kai Karos**

Belgium

**Jaro Karppinen**

Finland

**Alasdair Kay**

UK

**Defne Kaya**

Turkey

**Cemil Kayali**

Turkey

**Jenny Keating**

Australia

**Ishwara Keerthi**

India

**Alexis Kelekis**

Greece

**Bayram Kelle**

Turkey

**Simon Kelley**

Canada

**Stephen Kelly**

UK

**Friederike Kendel**

Germany

**Norelee Kennedy**

Ireland

**Oran Kennedy**

USA

**Peter Michael Kent**

Denmark

**Robert Kerslake**

UK

**Armin Keshmiri**

Germany

**Wasim Khan**

UK

**Piya Kiatisevi**

Thailand

**Megan Killian**

USA

**Uta Kiltz**

Germany

**Hunkyung Kim**

Japan

**Tae-Hwan Kim**

Korea, South

**Philip Kinghorn**

UK

**Sarah Kingsbury**

UK

**Chlodwig Kirchhoff**

Germany

**Renata Kirkwood**

Canada

**Rita Kiss**

Hungary

**Ali Kitis**

Turkey

**Andrew Kittelson**

USA

**Ydo Kleinlugtenbelt**

Netherlands

**Lars Klimaschewski**

Austria

**Jakob Klit**

Denmark

**Peter Kloen**

Netherlands

**Matthias C.M. Klotz**

Germany

**Beat Knechtle**

Switzerland

**Patrick Knott**

USA

**Cheryl Knudson**

USA

**Jih-Yang Ko**

Taiwan

**Gerjo Kok**

Netherlands

**Morey Kolber**

USA

**Sami Kolta**

France

**Yawei Kong**

USA

**Sk Kong**

Hong Kong

**Alice Kongsted**

Denmark

**Ljubica Konstantinovic**

Serbia

**Panagiotis Korovessis**

Greece

**Tomasz Kotwicki**

Poland

**Sandra Kraljevic Pavelic**

Croatia

**Roman Krawetz**

Canada

**Stefan Kroppenstedt**

Germany

**Sami Küçüksen**

Turkey

**Damien Kuffler**

Puerto Rico

**P. Paul F.M. Kuijer**

Netherlands

**Roel Kuijer**

Netherlands

**Ken Kumagai**

Japan

**Kensuke Kume**

Japan

**Chang-Fu Kuo**

UK

**Marie-Noelle Labour**

Ireland

**William Lack**

USA

**Christoph Ladel**

Germany

**Kuo-An Lai**

Taiwan

**Katri Laimi**

Finland

**Lisa Langsetmo**

Canada

**Rosanne Lanting**

Netherlands

**Malinee Laopaiboon**

Thailand

**Robert Laprade**

USA

**Pierre Lascombes**

Switzerland

**Laura Laslett**

Australia

**Richard Lass**

Austria

**Rick Lau**

Canada

**Thomas Läubli**

Switzerland

**Henrik Hein Lauridsen**

Denmark

**Carlos Lavernia**

USA

**Dana Lawrence**

USA

**Thomas Le Corroller**

France

**Benoit Le Goff**

France

**Ernesto Cesar Leal-Junior**

Brazil

**Andrew Leaver**

UK

**Charlotte Leboeuf-Yde**

Denmark

**Nam-Ki Lee**

Korea, South

**Young-Kyun Lee**

Korea, South

**Burkhard Leeb**

Austria

**Peter Leggat**

Australia

**Laurence Legout**

France

**Guang-Hua Lei**

China

**Brent Leininger**

USA

**Päivi Leino-Arjas**

Finland

**Marjatta Leirisalo-Repo**

Finland

**Jean Lemoyne**

Canada

**Erik Lenguerrand**

UK

**Lawrence Lenke**

USA

**Valentina Leone**

UK

**Ying Ying Leung**

Singapore

**Frankie Leung**

Hong Kong

**Victor Leung**

Hong Kong

**Marc Levesque**

USA

**Pazit Levinger**

Australia

**Ofer Levy**

UK

**Kang Li**

USA

**Tao Li**

China

**Linda Li**

Canada

**Hui Li**

China

**Xinning Li**

USA

**Jen-Chung Liao**

Taiwan

**Alexander Liddle**

UK

**Falk Liebers**

Germany

**Hong Chul Lim**

Korea, South

**Chuwen Lin**

USA

**Chung-Wei Christine Lin**

Australia

**Jinn Lin**

Taiwan

**Stefano Lipia**

Italy

**Gina Lisignoli**

Italy

**Hwa-Chang Liu**

Taiwan

**Jiaming Liu**

China

**Tang Liu**

China

**Yang Liu**

UK

**Yi Liu**

China

**Stefan Lohmander**

Sweden

**Rafael Lomas-Vega**

Spain

**Andrea Lopes**

Brazil

**Pedro A. Lopez-Miñarro**

Spain

**Mattia Loppini**

Italy

**Norailys Lorenzo**

Cuba

**Marcos Loreto Sampaio**

Canada

**Iraj Lotfinia**

Iran

**John Loughlin**

UK

**Anna Lowe**

UK

**X. Lucas Lu**

USA

**Anne Lubbeke**

Switzerland

**Daniel Lubelski**

USA

**Riccardo Luchetti**

Italy

**Jin Luo**

UK

**Jiaquan Luo**

China

**Zong-Ping Luo**

China

**Hannu Luomajoki**

Switzerland

**Kevin Lutsky**

USA

**Jörg Lützner**

Germany

**Mary Lynch**

Canada

**Xinlong Ma**

China

**Joy Macdermid**

Canada

**Luciana Macedo**

Canada

**Gustavo Machado**

Australia

**George Macheras**

Greece

**Ole Rintek Madsen**

Denmark

**Antonio Maestro**

Spain

**Laurence Magder**

USA

**Bruno Magnan**

Italy

**David Magne**

France

**John Magnussen**

Australia

**Petra Magosch**

Germany

**Suzanne Maher**

USA

**Asmaa Mahmoud Ali Moustafa**

Egypt

**Michele Maiers**

USA

**Mohammad Makki An**

USA

**Melih Malkoc**

Turkey

**João Mano**

Portugal

**Alfonso Manzotti**

Italy

**Luca Marchese**

Italy

**Kenneth Marcu**

USA

**Sven Märdian**

Germany

**Paul Marshall**

Australia

**Ramon Martinez**

Germany

**Toru Maruyama**

Japan

**Aidin Masoudi**

USA

**Yuichiro Matsui**

Japan

**Takashi Matsushita**

Japan

**Tobias Mattei**

Brazil

**Marco Matucci Cerinic**

Italy

**Georg Matziolis**

Germany

**Martin Maurer**

Switzerland

**Andreas Mavrogenis**

Greece

**Stephen May**

UK

**Eckart Mayr**

Germany

**Jenny Mcconnell**

Australia

**Lance Mccracken**

UK

**Suzanne Mcdonough**

UK

**Jane Mceachan**

UK

**Daniel Mcwilliams**

UK

**Francisco Medel**

Spain

**Geert Meermans**

Belgium

**Wolf Mehling**

USA

**Hitesh Mehta**

USA

**Jiong Mei**

China

**Matthew Menon**

Canada

**C Meskers**

Netherlands

**Cheryl Metcalf**

UK

**Ingrid Meulenbelt**

Netherlands

**Martina Michaelis**

Germany

**Michael Mienaltowski**

USA

**Magdi Mikhael**

UK

**Montserrat Mila**

Spain

**Freeman Miller**

USA

**Fábio Minozzo**

Canada

**Hermes Miozzari**

Switzerland

**Devyani Misra**

USA

**Toshiki Miura**

Japan

**Shigeru Miyaki**

Japan

**Takeshi Miyamoto**

Japan

**Hideaki Miyamoto**

Japan

**Vyvienne Roseline Piwai M'Kumbuzi**

Malawi

**Ali Mobasheri**

UK

**Hitesh Modi**

India

**Dalia Mohamed Ezz El Din El Mikkawy**

Egypt

**Mohd Shahrir Mohamed Said**

Malaysia

**Negin Mohtasham**

Iran

**Teemu Moilanen**

Finland

**Jamal Moiz**

India

**Niamh Moloney**

Australia

**Michael A. Mont**

USA

**Diego Montano**

Germany

**Eurico Monteiro**

Portugal

**Andrew Moore**

UK

**Paul Jarle Mork**

Norway

**Alessandro Muda**

Italy

**José Francisco Muñoz-Valle**

Mexico

**Shannon Munteanu**

Australia

**Bernadette Murphy**

Canada

**Saravanan Murugan**

India

**Giuseppe Musumeci**

Italy

**Stephanie Muth**

USA

**Corrie Myburgh**

Denmark

**Florian D. Naal**

Switzerland

**Takashi Nagai**

USA

**Satoshi Nagoya**

Japan

**Toshiyasu Nakamura**

Japan

**Yasuharu Nakashima**

Japan

**Surena Namdari**

USA

**Toshihiro Nanki**

Japan

**Sameer Naranje**

USA

**Michael Nasr**

USA

**Bård Natvig**

Norway

**Geraldine Naughton**

Australia

**Justine Naylor**

Australia

**Ara Nazarian**

USA

**Hossein Negahban**

Iran

**Giulio Negrini**

Italy

**Julie Neumann**

USA

**Bernard Ng**

USA

**Tuan Nguyen**

Australia

**Barbara Nicholl**

UK

**Leslie Nicholson**

Australia

**Mandy Nielsen**

Australia

**Mika Niemeläinen**

Finland

**Dejan Nikolic**

Serbia

**Hidetaka Nishida**

Japan

**Daniele Noel**

France

**Peter Nolte**

Netherlands

**Mike Notohamiprodjo**

Germany

**Wendy Novicoff**

USA

**Masahiko Nozawa**

Japan

**Soren O`Neill**

Denmark

**Ben Ockert**

Germany

**Neil O'Connell**

UK

**Ling Oei**

Netherlands

**Peter Oesch**

Switzerland

**Ralf Oheim**

Germany

**Naomi Oizumi**

Japan

**Matthew Olaogun**

Nigeria

**Francesco Oliva**

Italy

**Claudia Oliveira**

Brazil

**Vinicius Cunha Oliveira**

Brazil

**Patrick Omoumi**

Switzerland

**Ana-Maria Orbai**

USA

**Michelle Orme**

UK

**Nada Orsolic**

Croatia

**Georg Osterhoff**

Switzerland

**Andrew Östör**

UK

**Kieran O'Sullivan**

Ireland

**Slah Ouerhani**

Tunisia

**Tom Overend**

Canada

**Namik Kemal Ozkan**

Turkey

**Verity Pacey**

Australia

**Geert Pagenstert**

Switzerland

**Shea Palmer**

UK

**Vinod Panchbhavi**

USA

**Marco Paoloni**

Italy

**Francesco Paparo**

Italy

**Przemyslaw Paradowski**

Poland

**Jon Park**

USA

**Karel Pavelka**

Czech Republic

**Imre Pavo**

Austria

**Piero Pavone**

Italy

**E Scott Paxton**

USA

**Jennifer Paxton**

UK

**Ugo Pazzaglia**

Italy

**Mark Pearson**

UK

**George Peat**

UK

**Alma Becic Pedersen**

Denmark

**Clayton Peimer**

USA

**Paul Peloso**

USA

**Peter Pennekamp**

Germany

**Miguel Perez-Viloria**

USA

**Thomas Perneger**

Switzerland

**Carlo Perricone**

Italy

**Serge Perrot**

France

**Anthony Perruccio**

Canada

**Wolf Petersen**

Germany

**Mark Peterson**

USA

**Luzia Pfeifer**

Brazil

**Jonathan Phillips**

UK

**Susan Picavet**

Netherlands

**Eleonora Piccirilli**

Italy

**Gerald Pierard**

Belgium

**Rafael Pinto**

Australia

**Christian Plaass**

Germany

**Lilian Plotkin**

USA

**Spiros Pneumaticos**

Greece

**Zenon Pogorelic**

Croatia

**Michal Polguj**

Poland

**Chatlert Pongchaiyakul**

Thailand

**Albrecht Popp**

Switzerland

**Keith Porter**

UK

**Franco Postacchini**

Italy

**Klaas Postema**

Netherlands

**Esther Potier**

France

**Maria Prat**

Italy

**Arthur Pratt**

UK

**Martin Pressman**

USA

**James Prior**

UK

**Tamir Pritsch**

Israel

**Karl-Josef Prommersberger**

Germany

**Thomas Pufe**

Germany

**Laura Punnett**

USA

**Peter Pushparaj**

Saudi Arabia

**Guixing Qiu**

China

**Cunye Qu**

USA

**Robin Queen**

USA

**Krishnakumar R**

India

**Erik Rader**

USA

**Marsha Raebel**

USA

**Eugene Rameckers**

Netherlands

**Roberta Ramonda**

Italy

**Jonas Ranstam**

Sweden

**Michael J. Raschke**

Germany

**Sten Rasmussen**

Denmark

**Eva Rasmussen Barr**

Sweden

**Mohammad-Reza Rasouli**

Iran

**Bjoern Rath**

Germany

**Akhilesh Rathi**

India

**Michael Skovdal Rathleff**

Denmark

**Janez Ravnik**

Slovenia

**Jennifer Read**

UK

**Frances Rees**

UK

**Jonathan Reeser**

USA

**Jan Reinhardt**

Switzerland

**Michiel Reneman**

Netherlands

**Oliver Rettig**

Germany

**Fabien Ricard**

France

**Brent Richards**

Canada

**Jim Richards**

UK

**Pascal Richette**

France

**Rv Rikard**

USA

**Jochen Ringe**

Germany

**Bernhard Rintelen**

Austria

**Jody Riskowski**

UK

**Fernando Rivadeneira**

Netherlands

**Hilde Stendal Robinson**

Norway

**James Robinson**

UK

**James Roche**

UK

**Stephen Roche**

South Africa

**Edward Roddy**

UK

**Eric Röhner**

Germany

**Ola Rolfson**

Sweden

**Ryan Ross**

USA

**Philippe Rosset**

France

**Barbara Rossi**

Italy

**Robert Rowell**

USA

**Jean-Sebastien Roy**

Canada

**Hannes Rudiger**

Switzerland

**Leonard Rudolf**

USA

**Karl Rudolphi**

Germany

**Pietro Ruggieri**

Italy

**Cormac Ryan**

UK

**Michael Ryan**

USA

**Simo Saarakkala**

Finland

**Luca Saba**

Italy

**Luigi Sabatini**

Italy

**Manuel Sabeti**

Austria

**Isabel Sacco**

Brazil

**Bahar Sadeghi Abdollahi**

Iran

**Surachai Sae-Jung**

Thailand

**Nilay Sahin**

Turkey

**Vasileios Sakellariou**

USA

**Francesco Sala**

Italy

**Stacie Salsbury**

USA

**Bryan Saltzman**

USA

**Jun San Juan**

USA

**Mikel Sánchez**

Spain

**Olof Sandberg**

Sweden

**Gunther H. Sandmann**

Germany

**Raimon Sanmarti**

Spain

**Brandon Santoni**

USA

**Marcio Santos**

USA

**Ricardo São Simão**

Portugal

**Rim Sassi**

Tunisia

**Tsuyoshi Sato**

Japan

**Christos Savopoulos**

Greece

**Olga Savvidou**

Greece

**Aik Saw**

Malaysia

**Nicola Saywell**

New Zealand

**Laura Scaramuzzo**

Italy

**Frederieke Schaafsma**

Australia

**Andrea Schaller**

Germany

**Hanna Schell**

Germany

**Jasper Schellingerhout**

Netherlands

**T Schepers**

Netherlands

**Ernestina Schipani**

USA

**Hagen Schmal**

Germany

**Bernhard Schmidt-Rohlfing**

Germany

**Florian Schmidutz**

Germany

**Andreas Schmitt**

Germany

**Michael Schnabel**

Germany

**Craig Schulz**

USA

**Carlo Alberto Scir&Egrave**

Italy

**Luca Maria Sconfienza**

Italy

**David Scott**

UK

**Whitney Scott**

UK

**Leyla Sedighipour**

USA

**Frank Seehaus**

Germany

**Francesco Segreto**

Italy

**Roberto Seijas**

Spain

**Ali Seker**

Turkey

**Timothy Sell**

USA

**Jonathan Sembrano**

USA

**Alex Senchenkov**

USA

**Gerhard Sengle**

Germany

**Eric Senneville**

France

**Na Jin Seo**

USA

**Gernot Seppel**

Germany

**Sabah Servaes**

USA

**Philipp Sewerin**

Germany

**Li Shangfu**

China

**Irving Shapiro**

USA

**Julia Shelton**

UK

**Haihong Shen**

Korea, South

**Hiroaki Shima**

Japan

**Mootaz Shousha**

Germany

**Gabriele Siciliano**

Italy

**Sebastian Siebenlist**

Germany

**Rafael Sierra**

USA

**Siddhartha Sikdar**

USA

**Mario Silva**

Italy

**F. Joseph Simeone**

USA

**Paolo Simoni**

Belgium

**David R. Sinacore**

USA

**Jasvinder Singh**

USA

**Kern Singh**

USA

**Cassian Sitaru**

Germany

**Tracy L. Skaer**

USA

**Oeystein Skare**

Norway

**Richard Skolasky**

USA

**Emmanouil Skordilis**

Greece

**Soren Thorgaard Skou**

Denmark

**David Slattery**

Australia

**Frank Sloan**

USA

**Gerard Slobogean**

Canada

**Derek Smith**

Australia

**Kirk Smith**

USA

**Toby Smith**

UK

**Dragica Smrke**

Slovenia

**Lynn Snyder-Mackler**

USA

**Remko Soer**

Netherlands

**Tuulikki Sokka**

Finland

**Eleni Solidaki**

Greece

**Enrique R. Soriano**

Argentina

**Danielle Southerst**

Canada

**Gwendolyn Sowa**

USA

**Antonio Spadaro**

Italy

**Dusko Spasovski**

Serbia

**Rebecca Stack**

UK

**Tasha Stanton**

Australia

**Walter Stanwood**

USA

**Andre Stark**

Sweden

**Lukas Staub**

Switzerland

**Ivan Steenstra**

Canada

**Manos Stefanakis**

Cyprus

**Ely Steinberg**

Israel

**Johann Steurer**

Switzerland

**Stefan Stich**

Germany

**Mette Jensen Stochkendahl**

Denmark

**Niki Stolwijk**

Netherlands

**Susan Stott**

New Zealand

**John Street**

Canada

**Marcus R Streit**

Germany

**Arnold Suda**

Germany

**Akihiro Sudo**

Japan

**Olaf Suess**

Germany

**Sandra Sulsky**

USA

**Wadym Sulyma**

Ukraine

**Hui Sun**

USA

**Michiro Susa**

Japan

**Michael Swain**

Australia

**Marek Synder**

Poland

**Elizabeth Szalay**

USA

**Pawel Szulc**

France

**Alberto Tagliafico**

Italy

**Ching-Lung Tai**

Taiwan

**Roland Takács**

Hungary

**Shuji Taketomi**

Japan

**Jun Takeuchi**

Japan

**Chee-Wee Tan**

UK

**Boonsin Tangtrakulwanich**

Thailand

**Ekamol Tantisattamo**

USA

**Kristin Taraldsen**

Norway

**Mark Tarnopolsky**

Canada

**Halil Taskin**

Turkey

**Paula Tavares**

Portugal

**Ruben Tavares**

Canada

**Benjamin Taylor**

USA

**Allison Taylor**

USA

**Andrew Teichtahl**

Australia

**Marco Teli**

Italy

**René Ten Broeke**

Netherlands

**Gabriel Tender**

USA

**Ulrich W Thomale**

Germany

**Ellen Thomas**

UK

**Gethin Thomas**

Australia

**Martin Thomas**

UK

**Andrew Thoreson**

USA

**Saket Tibrewal**

UK

**Thomas Tischer**

Germany

**Louise Tofts**

Australia

**Yasuaki Tokuhashi**

Japan

**Sarah Tom**

USA

**Maja Tomicic**

Croatia

**Christy Tomkins-Lane**

Canada

**Steven Tommasini**

USA

**Diana Toneva**

Bulgaria

**Daniel Tonge**

UK

**Yvan Torrente**

Italy

**Martin Torriani**

USA

**Satish Totey**

India

**Trifon Totlis**

Greece

**Julia Treleaven**

Australia

**Guy Trudel**

Canada

**Raymond Tsang**

Hong Kong

**Hiroshi Tsumura**

Japan

**Jan Tuckermann**

Germany

**Stefan Tukaj**

Germany

**Faik Türkmen**

Turkey

**Glen Turley**

UK

**Peter Turnpenny**

UK

**Luigi Uccioli**

Italy

**Kosuke Uehara**

Japan

**Akin Ugras**

Turkey

**Joseph Umunnah**

Nigeria

**Ewoud Van Arkel**

Netherlands

**Sebastiaan Van De Groes**

Netherlands

**Michiel Van De Sande**

Netherlands

**Cornelia Van Den Ende**

Netherlands

**Wilbert Van Den Hout**

Netherlands

**Sarita Van Geest**

Netherlands

**Hubertus Van Hedel**

Switzerland

**Albert Van Houten**

Netherlands

**Joyce Van Meurs**

Netherlands

**Jaap Van Netten**

Netherlands

**Carlijn Van Randeraad-Van Der Zee**

Netherlands

**Lilian Van Tuyl**

Netherlands

**Jan-Paul Van Wingerden**

Netherlands

**Carla Vanti**

Italy

**Stephan Vehmeijer**

Netherlands

**Pascal-André Vendittoli**

Canada

**Jan Verbruggen**

Netherlands

**Arianne Verhagen**

Netherlands

**J.J. Verlaan**

Netherlands

**Ann Beatrice Vernallis**

UK

**Barbara Vertel**

USA

**Annemieke Verwoerd**

Netherlands

**Peter Vestergaard**

Denmark

**Esther Vicente**

Spain

**Bill Vicenzino**

Australia

**Amanda Villalvilla**

Spain

**Ernest Vina**

USA

**Jonas Vinstrup**

Denmark

**Marijn Vis**

Netherlands

**Livia Visai**

Italy

**Michael Vives**

USA

**Esther Vögelin**

Switzerland

**C.J. Vos**

Netherlands

**Frank Vrionis**

USA

**Jens Wahlström**

Sweden

**Karen Walker-Bone**

UK

**Jennifer Walsh**

UK

**Nicole Walsh**

Australia

**David Walton**

Canada

**Benedict Wand**

Australia

**William Wang**

China

**Zili Wang**

China

**Jing Wang**

USA

**Xuejun Wang**

USA

**Yuanyuan Wang**

Australia

**Iftikhar Wani**

India

**Axel Wanivenhaus**

Austria

**Guido Wanner**

Switzerland

**Stuart Warden**

USA

**Mary Chester Wasko**

USA

**Sandra Webber**

Canada

**Patrick Weber**

Germany

**James Wei**

Taiwan

**Lars Weidenhielm**

Sweden

**Shimon Weitzman**

Israel

**Robert Werner**

USA

**Maria M. Wertli**

Switzerland

**Martin Wessling**

Germany

**Chris Whatman**

New Zealand

**Rod Whiteley**

Qatar

**J. Michael Wiater, Md**

USA

**Jessica Widdifield**

Canada

**Kurt Wiendieck**

Germany

**Cylie Williams**

Australia

**Frances Williams**

UK

**Bettina Willie**

Germany

**Neil Wilson**

UK

**Fiona Wilson**

Ireland

**Jan Winters**

Netherlands

**Tania Winzenberg**

Australia

**Barton Wise**

USA

**Johan Witt**

UK

**Anita Wluka**

Australia

**Jennifer Wolf**

USA

**Kc Wong**

Hong Kong

**Li-Dong Wu**

China

**Lang Wu**

USA

**Kaihua Xiu**

USA

**Renjie Xu**

China

**Jianyun Yang**

China

**Mingyuan Yang**

China

**Akihiro Yasoda**

Japan

**Chee-Seng Yee**

UK

**Jeng-Hsien Yen**

Taiwan

**Gillian Yeowell**

UK

**Chengqing Yi**

China

**Serbulent Yigit**

Turkey

**Caglar Yilgor**

Turkey

**Vanessa Yingling**

USA

**Hiroyuki Yoshihara**

USA

**Taher Yousri**

UK

**Dahai Yu**

UK

**Ai-Xi Yu**

China

**Stefano Zaffagnini**

Italy

**Fabio Zaina**

Italy

**Fahed Zairi**

France

**Agostino Zampa**

Italy

**Julia F. Zandt**

Germany

**Kourosh Zarghooni**

Germany

**Mehdi Zeinalizadeh**

Iran

**Yin-Ping Zhang**

China

**Jianying Zhang**

USA

**Jingzhou Zhang**

USA

**Weidong Zhang**

China

**Hongyu Zhang**

China

**Yin-Gang Zhang**

China

**Qun Zhao**

China

**Chen-Guang Zhao**

China

**Xing Zhao**

USA

**Naiquan Zheng**

USA

**Liying Zheng**

USA

**Ruofu Zhu**

China

**Qin Zhuo**

China

**Carmine Zoccali**

Italy

**Arthur Zürcher**

Netherlands

**Michael G. Zywiel**

Canada

